# Tuberculosis: The insidious threat that compromises health in post-Assad Syria

**DOI:** 10.1016/j.ijregi.2025.100697

**Published:** 2025-07-01

**Authors:** Emir Muvaffak, Salah Safadi, Mohammad Al-Abbas, Mazen Kherallah, Abdulselam Daif, Alfonso J. Rodriguez-Morales, Josette Najjar-Pellet, Hazem Bakleh, Mahmoud Karim, Zaher Sahloul, Aula Abbara

**Affiliations:** 1 Hamidiye International Faculty of Medicine, University of Health Sciences, Istanbul, Turkey; 2 Hand in Hand for Aid and Development, Gaziantep, Turkey; 3 Department of Critical Care Medicine, Sanford Health System, University of North Dakota, Fargo, USA; 4 Syria Relief and Development, Gaziantep, Turkey; 5 Faculty of Health Sciences, Universidad Científica del Sur, Lima, Peru; 6 Grupo de Investigación Biomedicina, Facultad de Medicina, Fundación Universitaria Autónoma de las Américas-Institución Universitaria Visión de las Américas, Risaralda, Colombia; 7 Fondation Merieux, Middle East and North Africa Office, Beirut, Lebanon; 8 Syrian Arab Red Crescent, Damascus, Syria; 9 Independent consultant, Damascus, Syria; 10 Department of Pulmonology and Critical Care, University of Illinois, Chicago, IL, USA; 11 Department of Infectious Diseases, Imperial College London, London, UK; 12 Syria Public Health Network, UK

**Keywords:** Tuberculosis, Conflict, Health system, Detainees, Latent TB

## Abstract

This perspective explores the state of tuberculosis (TB) after the prolonged conflict in Syria and fall of the regime in December 2024; we discuss key considerations in light of multiple competing health priorities within Syria’s borders and the recovering health system. During the conflict, the health system fragmentation under differing geopolitical control led to unequal access to TB prevention, diagnostics and management social determinants such as poverty, malnutrition, inadequate water and sanitation, and lack of proper shelter, along with risks associated with disadvantaged groups, including internally displaced people, detainees, former detainees, and rural communities, not only increase the risk of TB transmission and the activation of latent infections but also hinder active case finding. Tackling these risks requires re-establishing the National TB Program (NTP) across the country, which acts equitably across all geographical areas to identify new cases, support robust surveillance activities, ensure drug resistance is identified promptly, and monitor treatment. Leadership from the Ministry of Health and the World Health Organization, with support from other stakeholders e.g., humanitarian, civil society or private sector can support the NTP and optimize health worker education and referral pathways. Beyond this, addressing the social determinants, which contribute to TB in Syria, is an essential component of TB control in post-conflict Syria.

## Highlights

• Conflict is detrimental to tuberculosis (TB) control due to health system impacts.
• Particularly vulnerable populations include the forcibly displaced and former detainees.
• Re-establishing Syria’s national TB program is essential to monitor and control TB.
• Addressing social determinants in Syria is important to prevent further increases.
• Improving healthcare access, targeted screening and active case finding are key.

## Health care system and tuberculosis challenges in Syria

Tuberculosis (TB) thrives in health systems that are fragmented and weakened by conflict; Syria presents one of the starkest examples of this [1]. The targeted destruction of health care infrastructure, including diagnostic services, has significantly disrupted access to TB diagnosis and treatment in a country where drug-resistant TB and HIV is an emerging concern; this presents a health security risk not only within Syria but also outside of its borders [2]. The diagnosis and treatment of TB, particularly drug-resistant TB, requires quality-assured reference laboratories that can guide treatment and ensure high fidelity of treatment compliance to reduce the risk of further drug resistance development [3] and to support treatment outcomes. Unlike other infections, treatment can take months with risks of adverse drug reactions, impacting compliance; this requires a robust health system with clear follow-up plans, which is challenging should patients lack access to services or be frequently displaced [4]. During the conflict, which escalated after uprisings in March 2011, the health system was devastated, fragmented, and politicized with severe under-resourcing and corruption. The fall of the five decade old Syrian regime in December 2024 presents new challenges for its transitional government and health system as it recovers from almost 14 years of conflict.

A first step is to re-establish and re-form Syria’s National TB Program (NTP) to ensure equitable health coverage across Syria. For this to occur, we must acknowledge the politicization of Syria’s health system, such that it was fragmented with at least three subnational health systems functioning within its borders. Access for patients with suspected or confirmed TB was extremely challenging across all areas, with vast inequalities and diversion of aid, particularly by the former Syrian regime [5]. The official figures reported by the former Ministry of Health (MoH) suggested that TB rates showed a decline from around 21 per 100,000 in 2010 to 17 per 100,000 in 2023; however, the actual rates of TB are likely much higher due to underdiagnosis and underreporting [1,6]. In addition, these figures do not consider areas outside of the former regime's control. As such, though the NTP received extensive support from the World Health Organization (WHO) (EMRO) in Damascus, it mainly served areas previously under regime control. This left the former area designated as northwest Syria (sheltering 5-6 million) and areas in northeast Syria (around 3-4 million) outside of its programming [5].

In the former northwest Syria, the WHO EURO-led health cluster based in Gaziantep, Turkey collaborated with Syrian-led humanitarian organizations, including Hand in Hand for Aid and Development, Syria Relief and Development, Bahar organization and the Independent Doctors Association, to support TB diagnostics, contact tracing, and treatment [4,7]. After many years of delay, they established access to services, detecting over 2500 patients between 2019 and 2025, including 47 cases of multidrug-resistant TB [7]. Northeast Syria, as in other aspects of the health system, has faced neglect; efforts in 2018 to support diagnostics and treatment for TB fell after the Hasakah prison attacks in January 2022 and have struggled to be re-established since [8]. Of great concern for this is the number of untreated detainees from this prison with concerns regarding uncharacterized drug resistance. A positive recent step is that the authorities in charge of northeast Syria recently signed a deal to unify with the central authority in Damascus, including integrating all civilian institutions into the Syrian state. This development presents an opportunity to work towards enhancing the implementation of the NTP across all regions of Syria, marking a significant step towards a unified health care system [9].

## Conflict and the social determinants of TB

Syria’s conflict has devastated gains made in mortality and health in the preceding decades; it had also transitioned from communicable to non-communicable diseases as the leading causes of morbidity and mortality. However, the conflict has disrupted these gains with numerous vaccine-preventable disease outbreaks, such as measles and polio, vector-borne diseases such as leishmaniasis, and cholera re-emergence, in addition to the breakdown in TB control [10]. The collapse of the extended program of immunization and TB vaccination has been an essential driver in these outbreaks. Alongside this, the decimation of social determinants, including shelter, water and sanitation, nutrition, and poverty (affecting 90% of the population), is also a significant contributor. For TB, these determinants are closely associated with the susceptibility for infection, the reactivation of latent disease and poor outcomes [1]. At the population level, addressing these determinants is essential to TB control and improving the health of Syria’s population [11]. They are closely allied to health and cannot be ignored.

Access to services is also key for earlier diagnosis and treatment; an estimated 16.7 million in Syria remain need humanitarian assistance. These must be addressed in a country where around 40-60% of health facilities are non-functioning or only partly functioning [12]. As with other morbidities, improved access to primary care services (and associated diagnostics), particularly for rural communities, is key; without this, Syria will remain far from achieving the sustainable development goals on universal health coverage. Diagnostic delays will also lead to increased periods of infectivity, such that, given overcrowding and poor ventilation, particularly among internally displaced people (IDPs), TB can spread further [4]. Due to the recent and ongoing geopolitical changes, Syria’s population remains highly mobile, both for IDPs and returnees who may return permanently or temporarily, depending on the states of their homes and available services [9]. In addition, the economic situation could prevent patients from traveling to TB centers. This necessitates services such as mobile clinics and imaging that respond to these communities’ needs and contextualized approaches to contact tracing and active case finding. Without these, undiagnosed and potentially infectious TB cases will remain a public health threat and place individuals at risk of adverse outcomes.

## Former detainees

The risks of TB, particularly drug-resistant TB, are stark for the more than 1.2 million who have been through Syria’s prison system [13]. At the time the regime capitulated, the estimated number of detainees and forcibly disappeared persons was 136,614, most of whom were held without charge or for the crime of opposing the regime. Of these, 24,200 detainees, including women and children, were released from various infamous prisons and detention centers, though a significant number remain unaccounted for [14]. Torture, starvation, overcrowding, isolation, poor ventilation, and limited sunlight were the norm, providing conditions in which TB could spread, or latent TB could reactivate [13]. Infected individuals often faced delayed diagnoses due to poor or unavailable health care services in prisons. Even if TB treatment was made available, it may be underdosed, run out, or be shared among prisoners, increasing the risk of under-treatment and drug resistance [15]. Those who emerged from the former regime prisons are emaciated and hold both psychological and physical scars. Their holistic needs must be considered in their recovery and their particular clinical risks, including investigation for TB. As yet, support for this highly vulnerable population is limited. A January 2025 report covering five governorates and interviewing 2112 recently freed detainees found TB to be the highest self-reported disease, with 261 reporting symptoms [16].

Although the former regime prisons have been evacuated, the humanitarian crisis related to detention in Syria has yet to be resolved. The authorities in NES are still detaining more than 46,500 individuals—those claimed to be members or relatives of the Islamic State of Iraq and the Levant—in overcrowded conditions, suffering from shortages of food, clean water, and medical care. Reports have stated that several hundred detainees have died from malnutrition and TB [17]. Alongside the need for a political solution and transitional justice, holistic medical care, TB investigation, and treatment should be priorities.

## Re-establishing Syria’s national TB program

The need to address TB as a public health concern in Syria is urgent and should build on existing models of what has worked across Syria’s health systems and learn from what has not [1]. Since 2017, International Office of Migration (IOM) has implemented the Middle East Response (MER) program—aimed at addressing TB, HIV, and malaria—in six regional countries, including Syria, with a grant from the Global Fund. MER has supported the NTP in close collaboration with the WHO and the MoH in Syria. Utilizing this grant to enhance health system priorities could be crucial in re-establishing the NTP across Syria and emphasizing governance and coordination, financing and sustainability, and community engagement strategies, alongside other core activities [18]. Prevention through TB vaccination and holistic public health measures, which should include social determinants, is likely to have the most significant impact on the population [11]. Rebuilding Syria’s infrastructure would improve the social conditions in which the population can regain their agency in the wake of the conflict and reduce the overall risks of TB.

**Figure 1:**
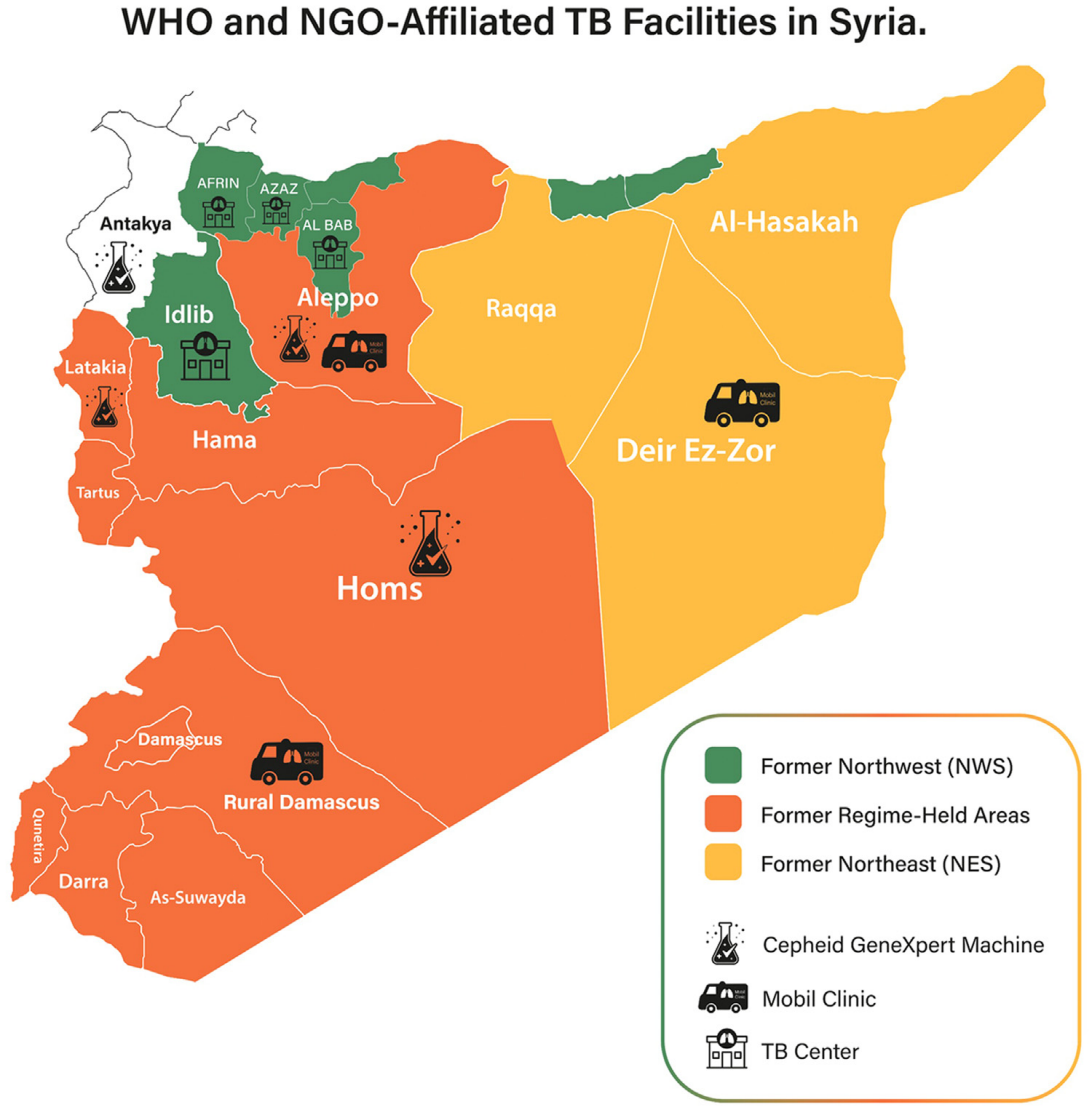
WHO and non-governmental organizations supported tuberculosis facilities. The WHO has installed 16 Cepheid GeneXpert machines in Latakia, Homs, and Aleppo. One additional machine in Antakya, Türkiye, was serving NWS. IN addition, the Middle East Response program has provided three mobile clinics, operated by the WHO, to support TB services, located in Aleppo, Rural Damascus, and Deir-ez-Zor. Four WHO-affiliated TB centers in former northwest Syria, covering Al-Bab, Azaz, Afrin, and Idlib. TB, tuberculosis; WHO, World Health Organization.

Beyond the MoH and the WHO-led NTP, humanitarian, civil society, and private actors can play a supportive role during Syria’s health system recovery; they can offer psychological, social, and economic support to patients and their families, particularly where the main earner is affected. In addition, public health campaigns that raise awareness, remove the stigma from patients with TB (including those co-infected with HIV), and sensitize both communities and health care professionals to the signs of TB are crucial to preventing missed cases. Education and training for all health care cadres are essential as a first step, with more tailored resources available to specialists, including doctors, TB nurses and pharmacists [1,7]. This should also include public health professionals focusing on screening and active case finding among particularly vulnerable groups, such as former detainees. For this to be successful, there needs to be a focus on strengthening primary care and referral pathways between the private sector, including clinics, laboratories, radiology centers, dispensaries, hospitals, and non-governmental organizations-affiliated TB facilities (Figure 1), and the MoH. This is key in monitoring dropouts and supporting their reintegration into the treatment program.

Decentralized access to diagnostics, particularly Cepheid GeneXpert machines, is key to supporting early detection and identification of the presence of drug resistance; this should include equitable distribution and access and ensuring that available machines are functioning; in 2023, the WHO provided 16 Cepheid GeneXpert machines, which were installed in Latakia, Homs, and Aleppo [19] with gaps present in northwest and northeast Syria; samples were then to be transferred to Syria’s national mycobacteriology laboratory in Damascus. However, as Syria’s health system is re-established, regional laboratories that are quality assured and can do phenotypic and genotypic identification of drug-sensitive and drug-resistant TB are needed. For better tracking of cases and monitoring, robust systems for reporting positive samples or cases commenced on TB treatment should be strengthened to more promptly identify clusters and outbreaks; this should include whole genome sequencing to support investigation [3].

Access to anti-TB therapy through the NTP, alongside rigorous monitoring, whether through directly observed therapy (DOT) or video observed therapy (VOT) to ensure compliance and monitoring, is essential, given the risks of drug resistance; the latter is potentially useful where there is adequate network coverage, particularly for those with long distances to travel. Related to this is the need for patient-held records, whether paper or electronic, such as a “health passport,” and strengthening networks across Syria. This would ideally include a unified health information or electronic health record system that health care providers can access; however, this requires some years of investment before it can be realized [20]. Given the current (often circular) mobility of Syria’s population and ongoing concerns around security, patient empowerment and education should be emphasized in the interim.

## Conclusion

Tackling TB in conflict and post-conflict settings such as Syria requires strong MoH and WHO NTP leadership with coordinated multi-agency efforts [20], particularly where there are ongoing population movements internally and across borders and where there is ongoing insecurity. The disparities in regional TB control efforts during the conflict should be assessed and integrated into a unified national program. Beyond the health care system, the social determinants, severely impacted by nearly 14 years of conflict, are closely linked to the TB burden. In addition, the risk for conflict victims, including released detainees, returnees, and IDPs, must be taken into consideration. To address these challenges, public health campaigns, education for healthcare professionals, supporting TB specialist nurses, targeted screening programs and active case finding are essential. These should be complemented by addressing social determinants and improving structural health system factors e.g. healthcare access including to diagnostics, patient held records/ tracking systems as essential components of an effective TB control strategy.

## Declarations of competing interest

The authors have no competing interests to declare.
